# Diffusion-Weighted MRI and Diffusion Kurtosis Imaging to Detect RAS Mutation in Colorectal Liver Metastasis

**DOI:** 10.3390/cancers12092420

**Published:** 2020-08-26

**Authors:** Vincenza Granata, Roberta Fusco, Chiara Risi, Alessandro Ottaiano, Antonio Avallone, Alfonso De Stefano, Robert Grimm, Roberta Grassi, Luca Brunese, Francesco Izzo, Antonella Petrillo

**Affiliations:** 1 Radiology Division, Istituto Nazionale Tumori–IRCCS-Fondazione G. Pascale, Napoli, Italia, Via Mariano Semmola, 80131 Naples, Italy; v.granata@istitutotumori.na.it (V.G.); a.petrillo@istitutotumori.na.it (A.P.); 2 Radiology Division, Universita’ Degli Studi Di Napoli Federico II, 80131 Naples, Italy; chiararisi@live.it; 3 Abdominal Oncology Division, Istituto Nazionale Tumori–IRCCS-Fondazione G. Pascale, Napoli, Italia, Via Mariano Semmola, 80131 Naples, Italy; a.ottaiano@istitutotumori.na.it (A.O.); a.avallone@istitutotumori.na.it (A.A.); a.destefano@istitutotumori.na.it (A.D.S.); 4 Siemens Healthcare GmbH, 91052 Erlangen, Germany; robertgrimm@siemens-healthineers.com; 5 Radiology Division, Universita’ Degli Studi Della Campania Luigi Vanvitelli, Piazza Miraglia, 80138 Naples, Italy; robertagrassi89@gmail.com; 6 Rector of the Universita’ Degli Studi Del Molise, 86100 Molise, Italy; luca.brunese@unimol.it; 7 Hepatobiliary Surgical Oncology Division, Istituto Nazionale Tumori–IRCCS-Fondazione G. Pascale, Napoli, Italia, Via Mariano Semmola, 80131 Naples, Italy; f.izzo@istitutotumori.na.it

**Keywords:** magnetic resonance imaging, DWI, DKI, liver metastasis

## Abstract

**Simple Summary:**

Imaging derived parameters can provide data on tumor phenotype as well as cancer microenvironment. Radiomics has recently shown potential in realizing personalized medicine. The aim of the manuscript is to detect RAS mutation in colorectal liver metastasis by Diffusion-Weighted Magnetic Resonance Imaging (DWI-MRI) - and Diffusion Kurtosis imaging (DKI)-derived parameters. We demonstrated that DKI derived parameters allows to detect RAS mutation in liver metastasis.

**Abstract:**

Objectives: To detect RAS mutation in colorectal liver metastasis by Diffusion-Weighted Magnetic Resonance Imaging (DWI-MRI) - and Diffusion Kurtosis imaging (DKI)-derived parameters. Methods: In total, 106 liver metastasis (60 metastases with RAS mutation) in 52 patients were included in this retrospective study. Diffusion and perfusion parameters were derived by DWI (apparent diffusion coefficient (ADC), basal signal (S0), pseudo-diffusion coefficient (DP), perfusion fraction (FP) and tissue diffusivity (DT)) and DKI data (mean of diffusion coefficient (MD) and mean of diffusional Kurtosis (MK)). Wilcoxon–Mann–Whitney U tests for non-parametric variables and receiver operating characteristic (ROC) analyses were calculated with area under ROC curve (AUC). Moreover, pattern recognition approaches (linear classifier, support vector machine, k-nearest neighbours, decision tree), with features selection methods and a leave-one-out cross validation approach, were considered. Results: A significant discrimination between the group with RAS mutation and the group without RAS mutation was obtained by the standard deviation value of MK (MK STD), by the mean value of MD, and by that of FP. The best results were reached by MK STD with an AUC of 0.80 (sensitivity of 72%, specificity of 85%, accuracy of 79%) using a cut-off of 203.90 × 10^−3^, and by the mean value of MD with AUC of 0.80 (sensitivity of 84%, specificity of 73%, accuracy of 77%) using a cut-off of 1694.30 mm^2^/s × 10^−6^. Considering all extracted features or the predictors obtained by the features selection method (the mean value of S0, the standard deviation value of MK, FP and of DT), the tested pattern recognition approaches did not determine an increase in diagnostic accuracy to detect RAS mutation (AUC of 0.73 and 0.69, respectively). Conclusions: Diffusion-Weighted imaging and Diffusion Kurtosis imaging could be used to detect the RAS mutation in liver metastasis. The standard deviation value of MK and the mean value of MD were the more accurate parameters in the RAS mutation detection, with an AUC of 0.80.

## 1. Introduction

Imaging derived parameters, when linked to other clinical data and correlated with outcome, can produce robust and accurate clinical decision support systems [1,2,3]. Imaging-derived parameters can be combined with genomics information in order to provide prognostic tools in oncological therapies. In fact, the various microRNA signatures expressions have been shown to correlate with treatment response, metastatic spread and prognosis [4,5,6]. Combined imaging-derived parameters and genomic signatures (“radio-genomic”) may be able to greatly enhance patient selection for different cancer therapy, predicting treatment response, addressing potential resistance to therapy, distinguishing favourable subsets of patients from those with poor prognosis and evaluating which patients may benefit from adjuvant therapy [3,7]. Various biomarkers have been identified for chemotherapy in advanced colorectal cancer (CRC). RAS and BRAF mutation and microsatellite instability status are considered significant biomarkers influencing medical oncologists’ decisions for systemic treatments. Considering the literature’s available data, RAS mutations analysis is important for anti-epidermal growth factor receptor (EGFR) therapy selection, and is deemed mandatory before treatment beginning in advanced CRC. In addition, CRC with wild-type RAS is not always sensitive to anti-EGFR antibodies, while BRAF-mutant CRC has a poor prognosis, as it is associated with lower chemotherapy sensitivity and with clinical situations particularly affecting patient’s performance status [8,9]. Kirsten-ras (KRAS) is an oncogene that forms an EGFR signaling cascade through various pathways, including Ras-RafMARK [10,11,12,13]. Through these pathways, KRAS modifies cell transformation and inhibits the tumor suppressor pathways. When KRAS mutations, which are found in 30–40% of CRC patients, are present, these pathways are activated continuously, which makes anti-EGFR monoclonal antibodies less effective in blocking EGFR [10,11,12,13]. Studies have shown that patients with KRAS-mutant tumors did not benefit from anti-EGFR antibody therapy [10,11]. Therefore, the presence of KRAS mutation in CRC is of great importance for the determination of individualized treatment. KRAS mutation is associated with morphologic tumor growth patterns. Moreover, increased cell growth activity induced by activated KRAS mutation seems to be essential for polypoid growth in CRC [14]. Then, the Diffusion-Weighted imaging (DWI) that offers functional quantitative information on the tissue’s microstructure by means of the water proton mobility differences and cellular density evaluation [15,16] can be used in the detection of RAS mutation. Water diffusion mobility is linked to cell density, vascularity and the viscosity of the extracellular apparent diffusion coefficient (ADC), and using a mono-exponential model or with diffusion and perfusion parameters in a bi-exponential model, it’s possible to individuate imaging biomarkers for fibrosis, tumor fluid and cell membrane integrity [17,18]. Using an Intravoxel Incoherent Motion method (IVIM) bi-exponential model to analyze DWI data, one can obtain the pure tissue coefficient (Dt) linked only to diffusion water mobility, the pseudo-diffusion coefficient (Dp) linked to blood mobility, and the perfusion fraction (fp) [19,20,21,22,23].

The traditional DWI data analysis approach is founded on the hypothesis that voxel water diffusion has a single component and follows a normal Gaussian distribution, and that water molecules diffuse without any constraint [23]. However, water molecule diffusion within biologic tissue exhibits non-Gaussian behavior [24]. Jensen et al. in 2005 reported a non-Gaussian diffusion model called Diffusion Kurtosis imaging (DKI) [24] used to analyze DWI data. This model includes the mean value of the kurtosis coefficient median (MK), which measures the tissue diffusion deviation from a Gaussian model, and the mean value of the diffusion coefficient (MD) with the correction of the non-Gaussian bias.

The aim of this study is to determine the potential of DWI- and DKI-derived parameters in the detection of RAS mutation in colorectal liver metastasis.

## 2. Materials and Methods

### 2.1. Dataset Characteristics

The National Cancer Institute of Naples institutional review board approved this retrospective study. The study was made in accordance with local relevant guidelines and regulations [21,22,25]. Informed consent considering the retrospective nature of the study was waived by the institutional review board. Each patient signed consent to data processing. We searched the radiological database at our institution from January 2018 to December 2019, and selected patients with colorectal liver metastases, who underwent MR study and hepatic resection. The inclusion criteria for the study population were as follows: (a) patients who had pathologically-proven liver metastases; (b) patients who had undergone MR imaging, with less than a 10–15 days interval by surgical resection; (c) availability of diagnostic quality pictures of the MR studies; and (d) availability of diagnostic quality pictures of the cut sections of the resected specimens in patients who underwent surgical resection for matching of imaging and pathology findings. The exclusion criteria were as follows: (a) conflict between the imaging-based diagnosis and the pathologically confirmed diagnosis; (b) no available MR images; and (c) no availability of contrast study.

In total, 66 patients with 126 liver metastases confirmed at pathology fulfilled the inclusion criteria during the study period. Among them, 14 patients were excluded for the following reasons: (a) 8 patients had no availability of diagnostic quality pictures of MR study; (b) 6 patients had no availability of contrast studies. Finally, 52 patients (25 women and 27 men; mean age, 59 years; range, 36–80 years) with 106 liver metastases comprised our study population. All liver metastases were analyzed. Among them 60 metastases with histologically confirmed RAS mutation were found. The characteristics of the patients and of metastases are summarized in Table 1. For each patient there was concordance between the presence of RAS mutation between primary tumor and metastasis.

### 2.2. MR Imaging Protocol

A 1.5 T Magnetic Resonance scanner (Magnetom Symphony, with Total Imaging Matrix Package, Siemens, Erlangen, Germany) with an 8-element body and phased array coils was used for MRI acquisition. MRI included basal images before intravenous (IV) injection of a hepato-specific contrast medium (CM) and then dynamic sequences obtained after IV injection of CM; the last series of images was acquired with a delay of 20 min during the CM hepatobiliary excretion. The MRI sequences fit the following criteria: coronal Trufisp T2-weighted free breathing; axial Half-Fourier Acquisition Single-Shot Turbo Spin-Echo (HASTE) T2-weighted, with controlled respiration, without and with fat-suppressed (FS) gradient-echo pulse; coronal HASTE T2-weighted, without FS; axial flash in-out phase T1-weighted, with controlled respiration; Volumetric Interpolated Breath-hold Examination (VIBE) T1-weighted Spectral adiabatic inversion recovery (SPAIR) with controlled respiration; DWI with echo-pulse planar sequence (EPI) at several b values (0, 50, 100, 200, 400, 600, 1000 and 2000 s/mm^2^). Details of sequence parameters were reported in Table 2 and in our previous manuscripts [1,25]. The MR sequences were acquired in free breathing. However, region of interest segmentation was performed avoiding encircling any distortion artefacts.

As liver-specific CM, the Gd-EOB-BPTA (Primovist, Bayer Schering Pharma, Germany), was employed—0.1 mL/kg of Gd-EOB-BPTA administrated using a power injector (Spectris Solaris^®^ EP MR, MEDRAD Inc., Indianola, IA, USA), at an infusion rate of 2 mL/s.

### 2.3. Data Analysis

Manual segmentation was performed by two expert radiologists, with at least 15 years of experience in MR liver imaging, drawing manually (and simultaneously avoiding encircling any distortion artefacts) the region of interests (ROIs) on diffusion-weighted imaging with the highest *b*-value, along the contours of the tumour to obtain a volume of interest (VOI) for each lesion [1,25]. DWI analyses were performed blinded to the clinical, RAS mutation and pathological data. No registration techniques were used to reduce movement artefacts, however VOI-based analysis was performed to reduce the influence of artefacts.

#### DWI Features

Voxel by voxel, by DWI 14 features were extracted using the mono-exponential model with all *b* values, the Diffusion Kurtosis imaging model with one low *b*-value 50 s/mm^2^ and multiple high *b*-values >200 s/mm^2^ (400, 600, 1000 and 2000 s/mm^2^) and the Intra-Voxel Incoherent Motion using a conventional bi-exponential fitting method based on the Levenberg Marquardt algorithm with multiple low *b*-values < 200 s/mm^2^ (0, 50, 100, 200 s/mm^2^) and higher *b*-values (400, 600 and 1000 s/mm^2^) [21,22,25].

The apparent diffusion coefficient (*ADC*) was obtained using the mono-exponential model [15,16]:

(1) $$ADC=\frac{ln\frac{S_{0}}{S_{b}}}{b}$$

where *S_b_* is the MRI signal intensity with diffusion weighting band, and *S*_0_ is the non-diffusion-weighted signal intensity.

In addition to the mono-exponential model, a conventional biexponential model using the Levenberg Marquardt fitting method was used to estimate the following IVIM-derived parameters: the pseudo-diffusivity (*D_p_* indicated also with D *), the perfusion fraction (*f_p_* indicated also with *f*), the tissue diffusivity (*D_t_*) and the basal signal *S*_0_:

(2) $$\frac{S_{b}}{S_{o}}=f_{p}\cdot \exp\left(-b\cdot D_{p}\right)+\left(1-f_{p}\right)\cdot \exp\left(-b\cdot D_{t}\right)$$

Diffusion Kurtosis imaging was included in the analysis in order to calculate Mean of Diffusion Coefficient (MD) and mean of Diffusional Kurtosis (MK) using Equation (3) by a two-variable linear least-squares algorithm [24]:

(3) $$\frac{S_{b}}{S_{o}}=\exp\left(-b\cdot D+\frac{1}{6}b^{2}\cdot D^{2}\cdot K\right)$$

In this equation, *D* is a corrected diffusion coefficient, and *K* is the excess Diffusion Kurtosis coefficient. *K* describes the degree that molecular motion deviates from the perfect Gaussian distribution. When *K* is equal to 0, Equation (3) evolves into a conventional mono-exponential Equation (1).

The parameters of conventional DWI (ADC), IVIM (FP, DT, DP) and DKI (MK and MD) were obtained per voxel by the prototype post-processing software Body Diffusion Toolbox (Siemens Healthcare, Erlangen, Germany).

Then, the mean and standard deviation values (STD) were calculated as representative values of each descriptor on ROI.

### 2.4. Statistical Analysis

Statistical analysis includes both univariate and multivariate approaches performed considering per-patient analysis.

#### 2.4.1. Univariate Analysis

The mean, median and standard deviation value (STD) and interquartile range were calculated as representative values of each descriptor among the group of patients with RAS mutation and without RAS mutation. Receiver operating characteristic (ROC) analyses were performed and the Youden index was used to individuate the optimal cut-off value for each feature. Considering the optimal cut-off values, area under ROC curve (AUC), sensitivity (SEN), specificity (SPEC), positive predictive value (PPV), negative predictive value (NPV) and accuracy (ACC) were calculated. The non-parametric Wilcoxon–Mann–Whitney U test for continuous variables was used for two-groups comparisons.

A *p* value < 0.05 was considered as significant. However, False Discovery Rate (FDR) adjustment according to Benjamini and Hochberg [26] for multiple testing was considered

The Statistics Toolbox of Matlab R2007a (MathWorks, Natick, MA, USA) was used for statistical calculations.

#### 2.4.2. Multivariate Analysis

Linear classifier, support vector machine, k-nearest neighbours (KNN) and decision tree were considered to assess the diagnostic accuracy using all DWI- and DKI-derived parameters [27]. A non-parametric method for selecting features with the goal of maximizing the prediction accuracy of classification algorithms was performed using the Neighbourhood component analysis (NCA). NCA feature selection with regularization to learn feature weights in order to minimize an objective function that measures the average leave-one-out classification or regression loss over the training was obtained by the Statistics and Machine Learning Toolbox™ function fscnca of Matlab [28].

Cross-validated using the leave-one-out validation approach and median values of AUC, accuracy, sensitivity and specificity were considered.

The Statistics and Machine Learning Toolbox of Matlab R2007a (MathWorks, Natick, MA, USA) was used.

## 3. Results

### 3.1. Univariate Analysis Results

Table 3 reports mean, median, standard deviation and interquartile range values for the extracted metrics with and without RAS mutation.

**Figure 1:**
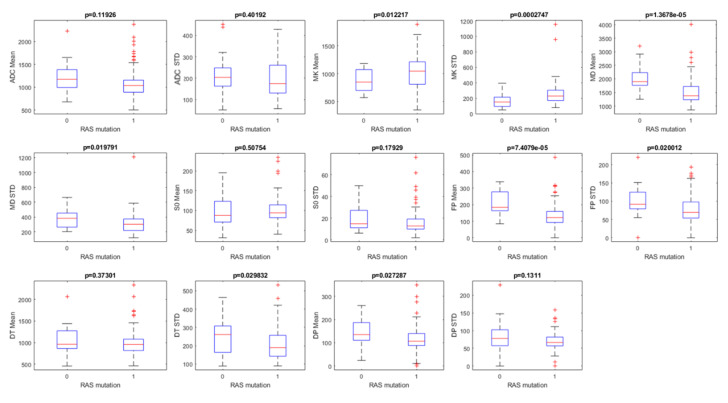
Boxplot of Apparent Diffusion Coefficient (ADC)-, Intravoxel Incoherent Motion method (IVIM)- and Diffusion Kurtosis Imaging (DKI)-derived parameters to differentiate presence/absence of RAS mutation.

Figure 1 reports the boxplot for diffusion extracted parameters to detect RAS mutation; a significant discrimination, considering the False Discovery Rate (FDR) adjustment, between the group with RAS mutation and the group without RAS mutation was obtained by the standard deviation value of MK (MK STD), by the mean value of MD and by mean value of FP.

**Figure 2:**
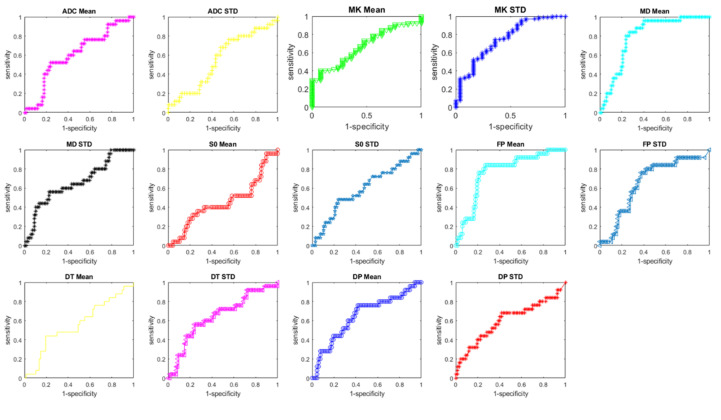
ROC curves of ADC-, IVIM- and DKI-derived parameters to differentiate presence/absence of RAS mutation.

Table 4 reports the diagnostic performance for ADC and for the extracted DWI (IVIM) and DKI features to detect RAS mutation. The best results were reached by MK STD with an AUC of 0.80 (sensitivity of 72%, specificity of 85%, PPV of 85%, NPV of 69%, accuracy of 79%) using a cut-off of 203.90 × 10^−3^, and by the mean value of MD with AUC of 0.80 (sensitivity of 84%, specificity of 73%, accuracy of 77%) using a cut-off of 1694.30 mm^2^/s × 10^−6^ (Figure 2).

**Figure 3:**
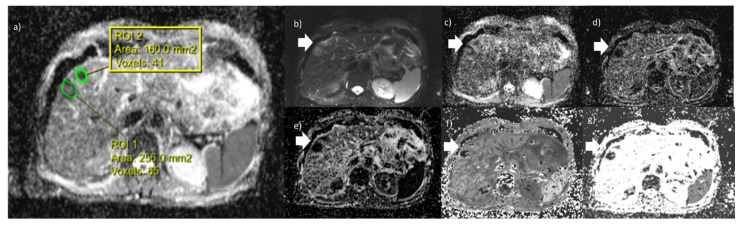
Representative case with two liver metastases and all DWI- and DKI-derived parameters maps: ADC map (**a**), S0 map (**b**), DT map (**c**), DP map (**d**), FP map (**e**), MK map (**f**), MD map (**g**).

Figure 3 shows a case with two liver metastases with all DWI- and DKI-derived parameter maps: ADC map (a), S0 map (b), DT map (c), DP map (d), FP map (e), MK map (f) and MD map (g).

### 3.2. Multivariate Analysis Results

**Figure 4:**
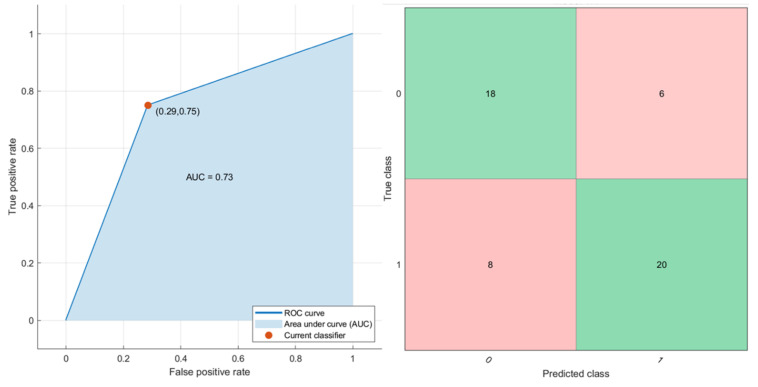
ROC curve and confusion matrix of the KNN tested considering all diffusion- and perfusion-extracted parameters by DWI and DKI.

**Figure 5:**
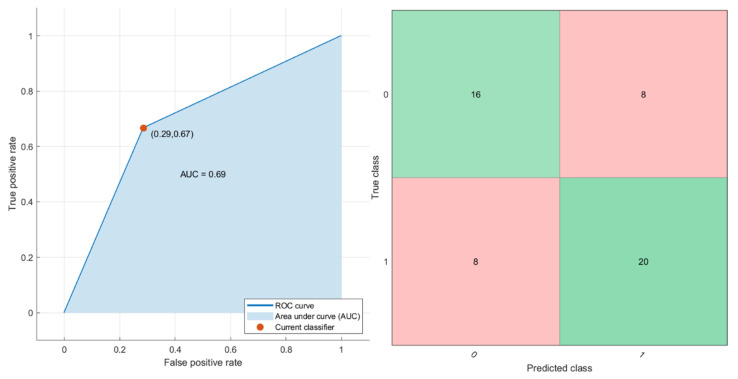
ROC curve and confusion matrix of the KNN tested considering the predictors derived by the feature selection procedure (mean value of S0, the standard deviation value of MK, FP and of DT).

Considering all extracted features, the best result was obtained considering a KNN that obtained an AUC = 0.73, with a sensitivity of 71%, a specificity of 75% and an accuracy of 73% (Figure 4). The classification training duration is 9.83 s. Considering NCA results, the features to use as predictors were the mean value of S0, the standard deviation value of MK and FP, and that of DT with respective feature weights of 0.41, 0.48, 0.47 and 0.49. Using these predictors, a KNN reached the best results with an AUC of 0.69, a sensitivity of 71%, a specificity of 67% and an accuracy of 73% (Figure 5). The classification training duration was 7.58 s. However, in both analyses, the tested pattern recognition approaches did not determine an increase in diagnostic accuracy to detect RAS mutation with respect to the single parameter.

## 4. Discussion

In this study we assessed Diffusion-Weighted MRI- and Diffusion Kurtosis imaging-derived parameters to detect RAS mutation in liver metastasis, showing a significant discrimination between the group with RAS mutation and the group without RAS mutation by the standard deviation value of MK (MK STD), by the mean value of MD and by the mean value of FP. The diagnostic performance for ADC and for the extracted DWI (IVIM) and DKI features in detecting RAS mutation using monovariate and multivariate analysis was evaluated. In the monovariate analysis, the best results were reached by MK STD and by the mean value of MD with an AUC of 0.80 (accuracy of 79% and 77% respectively). Instead, considering all extracted features or the predictors derived by the feature selection procedure (mean value of S0, the standard deviation value of MK, FP and of DT), the tested pattern recognition approaches did not determine an increase in diagnostic accuracy to detect RAS mutation with respect to the single parameter. To the best of our knowledge, this is the first paper that evaluates the correlation between IVIM and DKI parameters and RAS mutation in liver metastasis. Gültekin et al. [29] investigated whether there are any differences in apparent diffusion coefficient values obtained from colorectal liver metastases according to KRAS gene mutation status. In this retrospective study [29] were included 22 patients with 65 liver metastases due to colorectal cancer, and the patients were divided into two groups with KRAS mutation positive (+) (*n*: 10, 30 lesions) and the wild-type group (*n*: 12, 35 lesions). The lower ADC and ADC mean values were found to be statistically significantly lower in the KRAS (+) group compared to the wild-type group. ROC curve analysis revealed a statistically significant difference in terms of lower ADC and ADC mean with area under the curve (AUC) values of 0.680 and 0.760, respectively. Therefore, they concluded that the lower ADC and ADC mean values of colorectal liver metastasis are associated with the presence of KRAS mutation.

MRI quantitative analysis has recently shown potential in realizing personalized medicine for selecting the more appropriate therapy correlated with the different subtypes of tumor [29,30,31,32,33,34]. Several researchers assessed the imaging-derived parameter’s role as a precision medicine tool that may affect treatment strategies [29,30,31,32,33]. Oh et al. [31] used MR-based texture analysis and identified three imaging features that could differentiate mutant from wild-type KRAS. T2-weighted images could be used to predict KRAS mutation status preoperatively in patients with rectal cancer. To date, there are a limited number of studies on “radiogenomics” in liver neoplasms, mostly focused on hepatocarcinoma and cholangiocarcinoma [34,35,36,37,38].

Considering that a large rate of tumors were determined by mutations located in the MAPK pathway, KRAS and KRAS status should routinely be examined in combination with other clinic-pathological predictors in order to adapt optimal therapies and to stratify patient prognosis. In fact, the most significant finding related to KRAS-mutated tumors has been its ability to determine the anti-EGFR-based therapy’s eligibility. Moreover, KRAS mutations have been investigated for their potential to prognosticate recurrence and survival [39]. Data from the literature reported KRAS and BRAF mutations’ roles as prognostic and predictive biomarkers among patients undergoing colorectal cancer liver metastases hepatic resection [40,41]. In particular, KRAS and BRAF mutations were associated with worse overall survival and recurrence-free survival [40]. The relationship between radiomic parameters and RAS status offers advantages, allowing a better patient selection for cancer therapy, predicting treatment response, distinguishing favourable subsets of patients from those with poor prognosis, and evaluating which patients may benefit from surgical treatment.

This study had some limitations, as follows: data were derived from only one cancer center, with a single MR scanner; a small number of patients was considered, and this could influence the generalization of the conclusions; small sample size and use of Benjamini and Hochberg correction may lead to higher chances of type 2 error, and real effects might be overlooked; the retrospective nature of the study, and the absence of inter- and intra-reader variability and of a validation data set could affect the robustness of the results. Therefore, multicenter-perspective analyses, including more patients, inter- and intra-reader variability analysis and a validation data set are needed.

## 5. Conclusions

Diffusion-Weighted imaging and Diffusion Kurtosis imaging could be used to detect the RAS mutation in liver metastasis. The standard deviation value of MK and the mean value of MD were the more accurate parameters in the RAS mutation detection, with an AUC of 0.80; therefore, linked to other clinical data and correlated with outcome, if confirmed on a larger and different data set, these result could produce robust and accurate clinical decision support systems and tools for personalized medicine.

## Figures and Tables

**Table 1:** Characteristics of the study population.

	Patients with RAS Mutation	Patients with Wild-Type	All Patients
Patients Description	Numbers (%)/Range		
Gender	Men 15 (53.6%)	Men 12 (50.0%)	Men 27 (51.9%)
	Women 13 (46.4%)	Women 12 (50.0%)	Women 25 (48.1%)
Age	58 y; range: 36–79 y	59 y; range: 37–80 y	59 y; range: 36–80 y
Primary Cancer Site			
Colon	15 (53.6%)	13 (54.2%)	28 (53.8%)
Rectum	13 (46.4%)	11(45.8%)	24 (46.2%)
Hepatic Metastases Description			
Patients with single nodule	11 (39.3%)	9 (37.5%)	20 (38.5%)
Patients with multiple nodules	17 (60.7%)	15(62.5%)	32 (61.5%)/range: 2–15 metastases
Nodule size (mm)	mean size 35.2 mm; range 19–54 mm	mean size 33.7 mm; range 18–52 mm	mean size 34.4 mm; range 18–54 mm

**Table 2:** Pulse sequence parameters on MR studies.

Sequence	Orientation	TR/TE/FA (ms/ms/deg.)	AT (min)	Acquisition Matrix	ST/Gap (mm)	FS
Trufisp T2-W	Coronal	4.30/2.15/80	0.46	512 × 512	4/0	without
HASTE T2-W	Axial	1500/90/170	0.36	320 × 320	5/0	without and with (SPAIR)
HASTE T2-W	Coronal	1500/92/170	0.38	320 × 320	5/0	without
In-Out phase T1-W	Axial	160/2.35/70	0.33	256 × 192	5/0	without
DWI	Axial	7500/91/90	7	192 × 192	3/0	with (SPAIR)
Vibe T1-W	Axial	4.80/1.76/12	0.18	320 × 260	3/0	with (SPAIR)

Note: TR = Repetition time, TE = Echo time, FA = Flip angle, AT = Acquisition time, ST = Slice thickness, FS = Fat suppression, SPAIR = Spectral adiabatic inversion recovery.

**Table 3:** Mean, median, standard deviation value (STD) and interquartile range for each of the derived parameters in the group with RAS mutation and in the group without RAS mutation.

		ADC Mean [mm^2^/s × 10^−6^]	ADC STD [mm^2^/s × 10^−6^]	MK Mean [× 10^−3^] *	MK STD [× 10^−3^] *	MD Mean [mm^2^/s × 10^−6^]	MD STD [mm^2^/s × 10^−6^]	S0 Mean [*]	S0 STD [*]	FP Mean [%]	FP STD [%]	DT Mean [mm^2^/s × 10^−6^]	DT STD [mm^2^/s × 10^−6^]	DP Mean [mm^2^/s × 10^−5^]	DP STD [mm^2^/s × 10^−5^]
No RAS Mutation	Mean	1193.38	213.81	879.27	162.14	2017.08	374.18	97.56	20.34	202.42	96.34	1049.34	251.51	142.36	82.88
	Median	1174.40	203.80	846.60	152.60	1898.30	384.30	87.60	14.80	183.80	91.70	960.50	261.00	135.10	77.80
	Standard Deviation	327.82	94.99	193.49	84.38	463.51	123.39	36.53	12.48	72.61	46.11	324.33	90.55	59.89	50.25
	Interquartile range	417.50	91.45	381.40	122.95	475.20	195.05	55.05	17.30	121.15	46.35	417.65	154.25	82.65	47.40
RAS Mutation	Mean	1107.09	194.93	1025.01	259.83	1537.75	316.69	102.26	17.27	138.28	79.82	1010.55	211.69	116.72	68.60
	Median	1037.80	174.20	1044.50	229.50	1374.10	304.10	94.00	12.80	122.20	69.30	957.00	188.90	106.80	66.40
	Standard Deviation	373.93	80.87	304.05	166.81	559.97	152.54	39.32	13.17	80.12	43.09	342.71	91.55	60.31	30.11
	Interquartile range	267.30	130.70	406.20	135.00	495.40	159.70	33.10	9.70	70.10	44.30	277.10	116.10	52.30	25.10

Note: STD = standard deviation; ADC = Apparent Diffusion Coefficient; MD = mean of diffusion coefficient; fp = perfusion fraction; Dt = tissue pure diffusion; Dp = pseudodiffusion; * = dimensionless number.

**Table 4:** Diagnostic performance of ADC-, IVIM- and DKI-derived parameters to detect RAS mutation.

	AUC	Sensitivity	Specificity	PPV	NPV	Accuracy	Cut-off
**ADC Mean**	0.61	0.52	0.76	0.45	0.81	0.70	1157.41
**ADC STD**	0.56	0.76	0.45	0.34	0.83	0.53	166.70
**MK Mean**	**0.80**	**0.72**	**0.85**	**0.85**	**0.69**	**0.79**	**203.90**
**MK STD**	0.27	0.06	1.00	1.00	0.46	0.48	3218.51
**MD Mean**	**0.80**	**0.84**	**0.73**	**0.54**	**0.92**	**0.77**	**1694.30**
**MD STD**	0.66	0.56	0.78	0.48	0.83	0.72	377.11
**S0 Mean**	0.45	0.32	0.78	0.35	0.75	0.65	117.10
**S0 STD**	0.59	0.48	0.76	0.43	0.80	0.68	19.60
**FP Mean**	**0.77**	**0.84**	**0.73**	**0.54**	**0.92**	**0.75**	**148.30**
**FP STD**	0.66	0.76	0.63	0.43	0.88	0.66	79.10
**DT Mean**	0.56	0.44	0.81	0.46	0.79	0.71	1100.62
**DT STD**	0.65	0.56	0.76	0.47	0.82	0.71	258.40
**DP Mean**	0.65	0.76	0.58	0.40	0.87	0.63	113.60
**DP STD**	0.60	0.68	0.58	0.38	0.83	0.61	69.30

Note. AUC = are under curve; PPV = positive predictive value; NPV = negative predictive value. In bold are reported the significant parameters in the detection of RAS mutation.

## Abbreviations

ACC	accuracy
ADC	apparent diffusion coefficient
AUC	area under ROC curve
AT	Acquisition time
CRC	colorectal cancer
DP	pseudo-diffusion coefficient
DT	pure tissue coefficient
DWI	diffusion-weighted imaging
DKI	Diffusion Kurtosis Imaging
EPI	echo-pulse planar sequence
FA	Flip angle
FP	perfusion fraction
FS	fat-suppressed
HASTE	Half-Fourier Acquisition Single-Shot Turbo Spin-Echo
IVIM	Intravoxel Incoherent Motion method
KNN	K-Nearest Neighbor
MD	the mean value of diffusion coefficient with the correction of non-Gaussian bias
MK	mean value of kurtosis coefficient
MRI	Magnetic Resonance Imaging
NPV	negative predictive value
PPV	positive predictive value
ROI	Regions of interest
ROC	Receiver operating characteristic
SEN	sensitivity
SPAIR	Spectral adiabatic inversion recovery
SPEC	specificity
STD	standard deviation
ST	Slice thickness
TE	Echo time
TR	Repetition time
VIBE	Volumetric Interpolated Breath-hold Examination
VOI	Volume of interest
